# Subcutaneous *Dirofilaria repens* infection in an imported dog in Denmark

**DOI:** 10.1186/s13028-025-00820-x

**Published:** 2025-07-01

**Authors:** Kurt Buchmann, Per Walter Kania, Per Johansen

**Affiliations:** 1 Department of Veterinary and Animal Sciences, Faculty of Health and Medical Sciences, University of Copenhagen, Stigbøjlen 7, Frederiksberg C, DK-1870 Denmark; 2 Veterinary Clinic, Aakirkeby, Denmark

**Keywords:** Climate, Dog, Filarioids, Nematode, Zoonosis

## Abstract

**Background:**

The filarioid nematode *Dirofilaria repens* infects mainly dogs and is transmitted by vector mosquitoes when biting the definitive host. The parasite has mainly been reported from Eastern and Southern Europe, but during recent decades it has expanded its geographic range to some countries in Central and Northern Europe. Here, we report the finding of a fully mature female *D. repens* in a dog in Denmark.

**Case presentation:**

A female specimen of the filarioid nematode *Dirofilaria repens* (superfamily Filarioidea, family Onchocercidae) was isolated from a ruptured subcutaneous nodule in an 18 months old Border Collie on the Danish island of Bornholm. The dog was born in Italy, where it lived for the first 3 months of its life, whereafter it was imported to Denmark via Switzerland. Species diagnosis was based on molecular methods (Polymerase chain reaction PCR and sequencing of ribosomal DNA (rDNA, ITS) and mitochondrial DNA (mtDNA, COX1, NADH) in combination with morphometric characterization. The viviparous nematode was relatively small (total length 102 mm, broadest width 0.6 mm). It had a prominent uterus containing numerous eggs at different embryonation stages, some of which showed fully developed microfilariae.

**Conclusions:**

*Dirofilaria repens* was originally reported from Southern Europe and Asia, but during recent decades, it expanded its distribution area northwards, allowing autochthonous transmission to occur in Germany, Poland, Estonia, Latvia, Lithuania and Finland. The present report provides the first description from Denmark of a fully mature female worm in a dog imported from Italy. Known vectors include species of mosquitoes within the genera *Aedes*,* Anopheles Coquillettidia* and *Culex*, which are endemic in Denmark, reflecting the risk of future autochthonous transmission also in Denmark, where climatic conditions now allow larval development in the vectors. Although suspected to be an imported case, it cannot be excluded that the infection was contracted in Denmark.

## Background

The filarioid nematodes constitute a widespread and species rich group of worms infecting a range of host species. In most cases, vertebrates are definitive hosts, and invertebrates serve as intermediate hosts (vectors) for these parasites. The superfamily Filarioidea comprises at least four families (Filariidae, Setariidae, Mesidionematidae, Onchocercidae), each containing a series of genera. In the family Onchocercidae, one of the 36 genera is *Dirofilaria*, which is of veterinary and medical importance. *D. repens* infects mainly dogs, occasionally other carnivores such as foxes and wolves, and causes subcutaneous dirofilariosis [1, 2]. The species is zoonotic, and human cases in endemic areas are not rare [3–7]. The adult worms occupy sites in various organs and tissues, and following mating, viviparous females release microfilariae (L1) into the circulation [1, 2]. When the host is bitten, the microfilariae are ingested by mosquitoes, in which they penetrate from the gut into the Malphigian tubules via the haemocoel and molt twice (L1-L2-L3) to reach the infective third larval stage (L3) [8]. These infective larvae migrate to the mouth parts of the mosquito (genera *Anopheles*,* Aedes*,* Culex* and *Coquillettidia*) and are then transmitted when they bite the definitive mammalian host. The larvae migrate to the subcutaneous location, molt twice (L3-L4-L5) and develop into the adult stage [2]. The geographical distribution was originally restricted to southern Europe and Asia [9–12], with the Mediterranean region as the main area of occurrence in Europe [13–19], but during recent decades the parasite has expanded its geographic range to some countries in Central Europe [20], Ukraine [21], Russia [22] and even more northern areas of Europe. Many of the cases in the northern regions were assigned as imported infections (dogs, humans), but recently strong evidence of autochthonous transmission has been presented in Estonia, Latvia, Lithuania (2008–2012) [23, 24], Poland (2014) [25], Germany (2016) [6] and Finland (2017) [26]. Spread of infected vectors (by air traffic, container transports, wind spread) may be one way to increase the area of distribution of the parasite. However, in most cases imports of infected dogs may lead to establishment of the life cycle in new regions, if suitable vectors are present. Focus should be placed on the risk of further spread to Denmark, Sweden and Norway due to the known import of infected animals [24]. Recently, an imported Romanian dog was found infected with microfilariae in Denmark, diagnosed based on blood samples by use of molecular techniques (Polymerase Chain Reaction, PCR, and sequencing of ribosomal DNA sequences, rDNA) [27], but no adult worm has hitherto been isolated and described. Likewise, an imported human case was reported in Denmark, suggesting that the patient had been infected with *D. repens* in Greece [28], but the diagnosis was established by ELISA and was not based on isolation of any larval or adult worms. We here present a case from the Danish island of Bornholm. An imported Border Collie presented with a ruptured subcutaneous nodule, from which an adult female specimen of *D. repens* appeared. Morphometric and molecular data of the worm and a comparison with various geographic isolates are presented in this report.

## Case presentation

### Patient

An 18-month-old Border Collie (born in Italy where it lived for its first 3 months before being transferred via Switzerland to Denmark) was found by its owner to have a ruptured nodule on its belly, which attracted attention. A whitish thin worm was pulled out of the ulcer by the owner and delivered to the veterinary clinic in Aakirkeby (Island of Bornholm, Baltic Sea) 1 October 2024. The worm was then conserved in 70% ethanol and transported to the University of Copenhagen for further analysis. The dog owner was contacted twice during a 50 day period post-diagnosis in order to secure the health and well-being of the dog. However, the owner reported that no clinical signs had been observed, neither had additional subcutaneous nodules been observed.

### Morphometric diagnosis

**Fig. 1:**
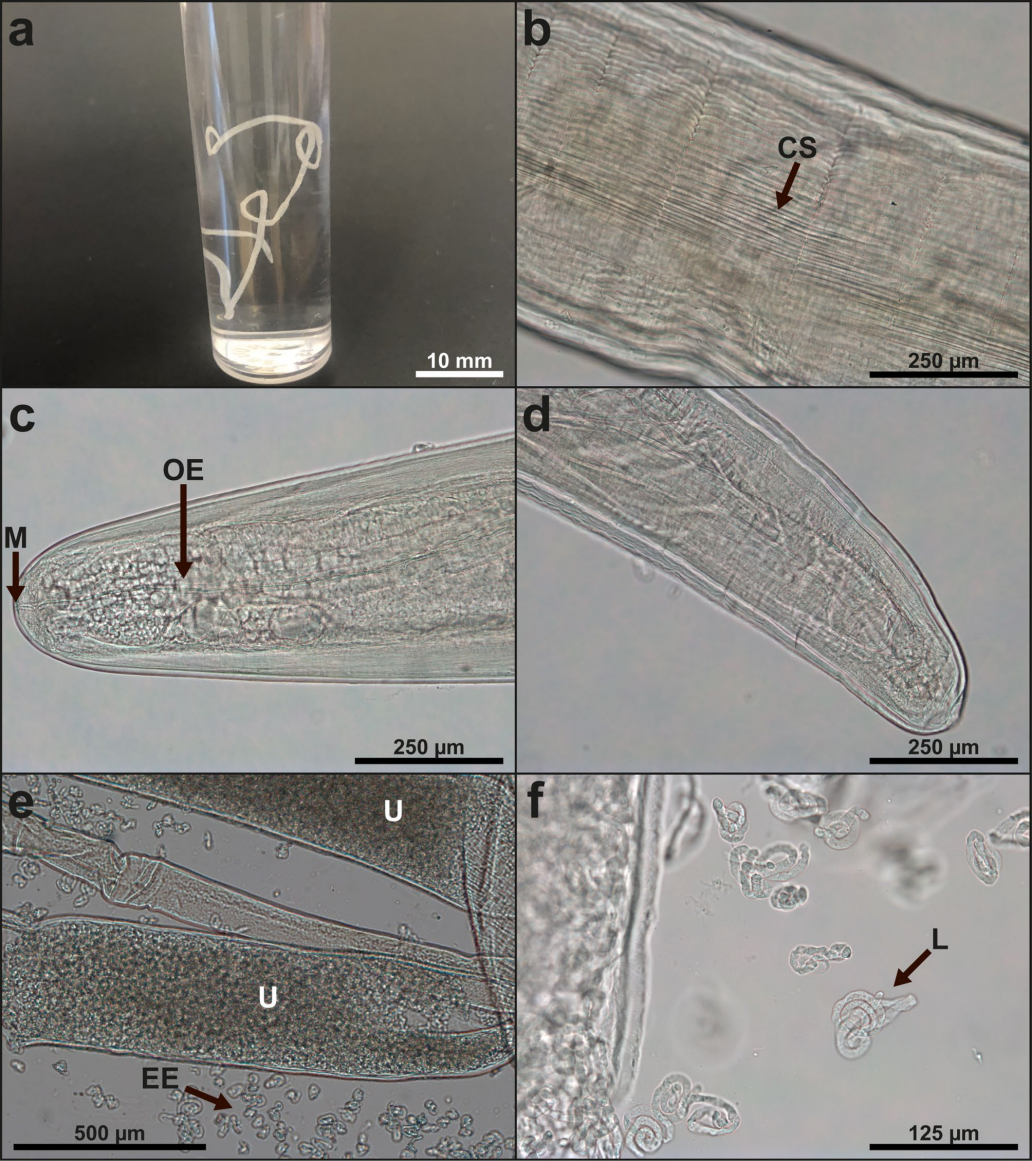
Morphological details of the Danish *Dirofilaria repens* isolate: **a.** Macroscopic view of worm in 96% ethanol following recovery from a ruptured subcutaneous nodule of a Border Collie., **b.** Middle part of worm with longitudinal rows of cuticular striations (ridges)., **c.** Cephalic end of worm., **d.** Caudal part of worm., **e**. Embryonated eggs prepared from the paired *D. repens* uterus. **f**. Microfilariae released from *D. repens* eggs. Abbreviations: CS: cuticular striations, M: mouth, OE: oesophagus, U: uterus, EE: embryonated eggs, L: larvae

Total length and width of the nematode were measured. Unstained sections of the anterior, middle and caudal parts were mounted in glycerine gelatine or lactophenol for microscopy [29]. Subsamples of the middle parts were kept unstained or were stained in Light Green (Sigma-Aldrich, Denmark) and mounted in mounting medium Aquatec (Merck, Germany). Measurements were conducted in the compound microscope (magnification 50-1000 X) (Leica DM5000B, Germany). One female specimen was identified (Fig. 1a), with cylindrical body shape, narrowing at both ends. The cuticle was up to 0.03 mm tick, carried longitudinal ridges and striations (Fig. 1b) and appeared segmented due to transverse indentations every 0.1 to 0.4 mm. The cephalic end was rounded and the caudal end obtuse (Fig. 1cd). The nematode measured 102 mm (total length of stretched worm) and 0.6 mm (broadest width). The cephalic end had a narrow circular mouth opening (Fig. 1c). The length of the oesophagus was 0.8 mm and the nerve ring was located 0.29 mm from the cephalic end. Width at cephalic end 0.2 mm, width at nerve ring level 0.3 mm, width at vulva level 0.5 mm, width at anus level 0.2 mm. The vulva was located below the junction of the short simple oesophagus with the intestine. The viviparous worm had a prominent uterus (Fig. 1e) containing numerous oocytes at different embryonation stages, some of which showed fully developed microfilariae (Fig. 1f). These morphometric data show compliance with the original description and redescriptions (Table 1).

**Table 1:** Morphometric analysis and diagnosis of the Danish *Dirofilaria repens* isolate (female specimen) compared to literature

Morphometric character	[10]	[11]	[12]	[35]	This study
Geographic origin	Italy	Vietnam	Vietnam	Poland	Denmark
Host species	Dog	Dog	Dog	Dog	Dog
Total length mm	100–170	100	106–131	147–161	102
Width (maximum) µm	460–650	460	410–510	490–570	600
Width (nerve ring level)		280		283–298	285
Width (anus level)		250		128–169	180
Width (vulva level) µm		370		499–508	500
Oesophagus length µm			820–953	915–1037	800
Oesophagus width (maximum) µm		100		77–102	101
Distance nerve ring- cephalic end			256–287	291–298	290
Distance vulva-cephalic end µm	1150–1620	1150	1580–1970	1445–1629	1500
Distance anus-caudal end µm	55–90	60			80
Embryon length µm	300–360	300–360			300–350

### Molecular diagnosis: Polymerase chain reaction (PCR) and sequencing

**Fig. 2:**
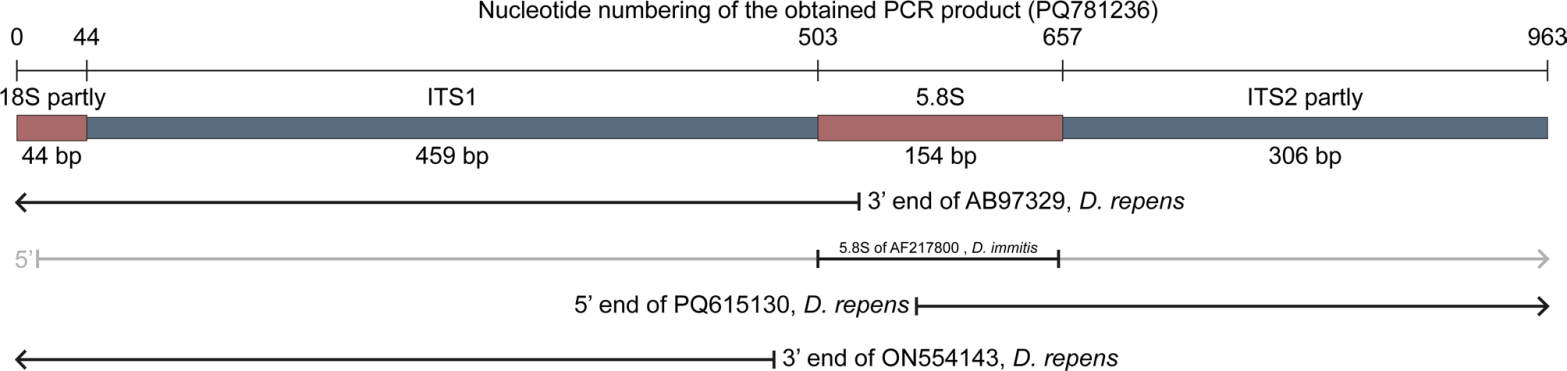
Graphical representation of the ITS region. A 963 bp long PCR fragment (submitted as PQ781236 at GenBank) was obtained and its features are outlined as boxes. Other GenBank entries related to PQ781236 are outlined using lines with indicated GenBank accession numbers and species names; arrow heads indicate the sequence continuous. Only the 5.8 S rRNA of AB97329 was used for comparison as the rest had rather low identity towards PQ781236

A 5 mm section of the middle part of the worm was placed in 96% ethanol and prepared for molecular diagnosis. Following evaporation of ethanol, the sample was exposed to proteinase K digestion, DNA purification and PCR amplifying rDNA (ITS) and mtDNA (COX1). Genomic DNA was purified using the QIAamp DNA Mini Kit (cat.no. 61306, Qiagen, Copenhagen, Denmark) according to the manufacturer’s instructions except that 50 µl elution buffer was used. PCR was performed in a BioRad T100 Thermal Cycler (cat.no. 1861096, Bio-Rad Laboratories, Copenhagen, Denmark) using volumes of 60 µl and 1 mM forward primer, 1 mM reverse primer (both synthesized at TAG Copenhagen, Denmark), 1 mM dNTP mix (dNTP Blend, cat.no. 10085714, Fisher Scientific, Roskilde, Denmark), 0.6 µl DNA Polymerase, 6 µl 10x Reaction buffer, 1.8 µl 50 mM MgCl_2_ (the last 3 included in BIOTAQ DNA Polymerase, cat.no BIO-21060, Saveen & Werner ApS, Jyllinge, Denmark). We used the following conditions for amplification of the internal transcribed spacer (ITS 1 and part of ITS 2 (including the 3’end of 18S, ITS1, 5.8S PCR, ITS2, and the 5’end of 28S) (Fig. 2): 1) pre-denaturation at 95°C for 5 min, 2) 40 amplification cycles of denaturation at 95°C for 30 s / annealing at 54°C for 1 min / elongating at 72°C for 1 min followed by post-elongation at 72°C for 7 min, 3) primers used for ITS were the forward primer BD1 (5’-GTCGTAACA AGGTTTCCGTA-3’) [30] and the reverse primer NC2 (5’-TTAGTTTCTTTTCCTCCGCT-3’) [31].

Amplification of the cytochrome c oxidase subunit 1 (COX1) applied the same PCR conditions except for annealing at 53°C for 105 s. Various primer combinations were found successful [32, 33] but in order to obtain a longer COX1 product we used the forward primer Drep2F (5’- GATTTATTTT TGTTATTTAA TATGAA-3’) and the reverse primer Ctc2R (5’ CCTTACTAAT AACCTTTCAA TGA-3’) [34]. The same PCR condition as for COX1 was used to amplify part of the mitochondrial gene NADH dehydrogenase subunit 1 (ND1) using the primers Nad1F (5’-TTGTAGTATG GTAGAGGTAA GG-3’) and Nd1R (5’-TTGTAGTATG GTAGAGGTAA GG-3’) [34]. The PCR products were visualized by 1.5% agarose gel-electrophoresis. Products were purified using the Illustra™ GFX™ PCR DNA and Gel Band Purification Kit (cat.no. 28-9034-71, VWR International A/S, Søborg, Denmark). Products were sequenced at Macrogen Europe (Amsterdam, Netherlands) and analyzed using the software CLC- Main Workbench v20.0.4. Multiple alignments and phylogenetic analyses were performed using the software CLC- Main Workbench v20.0.4. (QIAGEN, Copenhagen, Denmark). The two achieved sequences in this study were submitted to GenBank (NCBI) and obtained the accession numbers (PQ781236) (ITS) and (PQ772105) (COX1). Alignments were constructed in Clustall W, and model testing suggested that GTR + G + T was the best model. Maximum likelihood phylogeny was performed by first constructing a tree using the method UPGMA (100 bootstraps), and by using this as an initial tree we constructed a tree using the method Neighbor Joining (1000 bootstraps). In order to ensure proper alignment of the ITS1, the 3’ end of 18 S and 5’ ends of 5.8 S were included in the phylogenetic analyses (Fig. 2; Table 2). A recent phylogenetic *D. repens* analysis, based on 95 sequences, established 18 haplotypes (DR1 → DR18) based on combining two fragments of the COX1 (ON539990→ON540920) [34]. We performed a PCR using their primers and Ctc2F, which resulted in a 1621 bp long fragment exhibiting 99.94% identity with 100% coverage when compared to *D. repens* isolated from a human in Croatia (GenBank acc.no. KX265049). The phylogenetic COX1 study was based on combining two separate fragments [34]. Our study showed that these were separated by 171 bp. In order to compare our isolate with haplotypes presented [34] we established a similar construction. Performing a pairwise comparison against the 95 combined constructions [34] we found 100% identity to the 65 constructions designated as DR1, and we therefore assigned our new Danish isolate to haplotype DR1. These originated from a range of Central and East European countries (Latvia, Lithuania, Poland, Romania, and Ukraine). In addition, the ND1 gene was partly sequenced but was not useful for further subtyping.

**Table 2:** Description of genbank.entries covering the obtained parts of the ITS region. AB973229 ^A^ had a 3 bp long insertion between nucleotides between 264 and 265 of genbank.acc.no. PQ781236 (this study); excluding this insertion results in 100% identity. This insertion was absent in ON554143 ^b^. The nucleotides 535 to 566 of PQ781236 were not present in any *D. repens* entries in genbank. However, this part was covered by a nearby relative *D. immitis* (AF217800 ^c^); however, parts outside 5.8 S had very low identities and were not used for comparison. The parts of PQ781236 corresponding to other genbank.entries are indicated in the first column

Nucleotide no.	GenBank acc. no.	Species	Identity
1 → 534	AB973229 ^a^	*Dirofilaria repens*	99.43%
567 → 963	PQ615130	*Dirofilaria repens*	100%
1 → 481	ON554143 ^b^	*Dirofilaria repens*	100%
503 →659	5.8 S of AF217800 ^c^	*Dirofilaria immitis*	98.06%

The GenBank *D. repens* rDNA sequences available for comparison with our results were fragmented. Tables 2 and Fig. 2 describe the obtained rDNA covering 18S rRNA partly complete ITS1, complete 5.8S rRNA, and ITS2 partly. Some entries covered the 5’end of our sequences (e.g. ON554143, *D. repens* with 100% identity), others covered the 3’ end (e.g. PQ615130, *D. repens* with 100% identity). Thus a 32 bp long gap was present when performing BLAST search at NCBI. Our sequence covered the entire 5.8 S region as we detected and added the 32 bp long cap, which previously was missing.

**Fig. 3:**
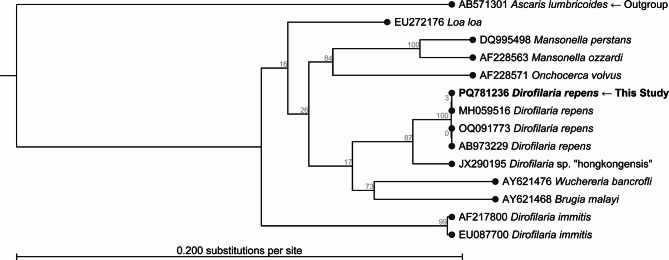
Phylogenetic tree of the Danish isolate of *D. repens* using ribosomal DNA (rDNA) covering 18 S partly, ITS1 complete, and 5.8 S partly

When the complete ITS1 (459 bp) sequence of *D. repens* achieved in this study was subjected to BLAST at GenBank the first 53 hits were *D. repens*. One had 100% coverage (OQ091773 from Denmark) with 99.4% identity. The remaining *D. repens* had 95% coverage and identities from 100 to 99.32%. When the ITS2 part (306 bp) of *D. repens* achieved in this study was subjected to BLAST at GenBank the first 36 hits were *D. repens*. The first 4 entries exhibited 100% identity and 100% coverage (GenBank accession numbers MK942385 (France), MN200338 (Russia: Voronezh), PQ615128 (Bosnia and Herzegovina) and PQ615130 (Bosnia and Herzegovina). The remaining 32 entries had at least 90.43% identity towards the one described in this study. The phylogenetic analysis (Fig. 3) supports the assignment of the achieved sequences to *D. repens*. No *D. repens* entries covered the complete 5.8 S rRNA. However, a close relative *D. immitis* (AF217800) covered the 5.8 S rDNA 100% with a total identity of 98.06%, but the gap of 32 bp was covered with 100% identity. The rRNA of our sequence (PQ781236) had a 3 bp deletion (between bp 264 and bp 265) as compared to some GenBank entries, e.g., ON554156 and AB973229. However, this deletion was apparent in e.g. ON554156 and 32 more sequences in a previous study [34]. This previous study established that absence / presence of this indel (insertion/deletion) is an indicator belonging to haplotype A and B, respectively. Therefore, the new Danish isolate PQ781236 was assigned to haplotype A with respect to the rRNA. In addition, we could confirm that a previously described *D. repens* from Denmark originating from a dog imported from Romania had the same 3 bp insert and was a haplotype A with respect to the rRNA [27]. The phylogenetic analysis of the 18 S partly, ITS1 complete, and 5.8 S partly rRNA (Fig. 3) supports the assignment of the achieved sequences to *D. repens*.

## Discussion and conclusions

The genus and species names for *Dirofilaria repens* was originally suggested in 1911 by the two French helminthologists, Railliet and Henry, in a report on filarioid parasites but without presenting a morphometric description [9]. In the same year, two other French researchers, Bauche and Bernard [11], published a detailed description of a filarioid parasite collected from a dog in Vietnam. Based on this publication, the Vietnamese isolate was declared to be identical to *D. repens* by Railliet and Henry [10], who based their comparative analysis on Italian dog isolates delivered from Bologna in Italy. They presented morphometric details allowing differential diagnosis towards the related heartworm *D. immitis*. Illustrated redescriptions of *D. repens*, including new collections in Vietnam and measurements of the original collections from 1911 of Bauche and Bernard, were presented 60 years later [12]. Corresponding relevant morphometric details of Polish isolates were reported from recent introductions in Poland [35]. It should be noted that different preparation techniques and mounting methods can influence morphometric characters to some extent. However, when comparing morphometric and morphological characters of the Danish isolate to data provided in the original descriptions, it was clearly indicated, that an adult female specimen of *D. repens* had been found in Denmark, although the length of the parasite (10.2 cm) was in the lower end of the expected range (10–17 cm). Molecular species confirmation was then conducted by PCR of the parasite DNA and the sequencing of rDNA (comprising ITS1, 5.8 S and part of ITS2) and mitochondrial DNA (COX1 and NADH). The new Danish isolate was further assigned the haplotypes DR1 (mtDNA) and A (rRNA). Full compliance (100% similarity) to European isolates of the species was found for mtDNA. However, some minor variations were shown with regard to parts of the rDNA sequences. It is unknown if these have any biological significance, but it may be speculated if these minor genetic differences are associated with other genetic changes in the isolate, with importance for pathogenicity and ability to establish in new geographic areas.

**Fig. 4:**
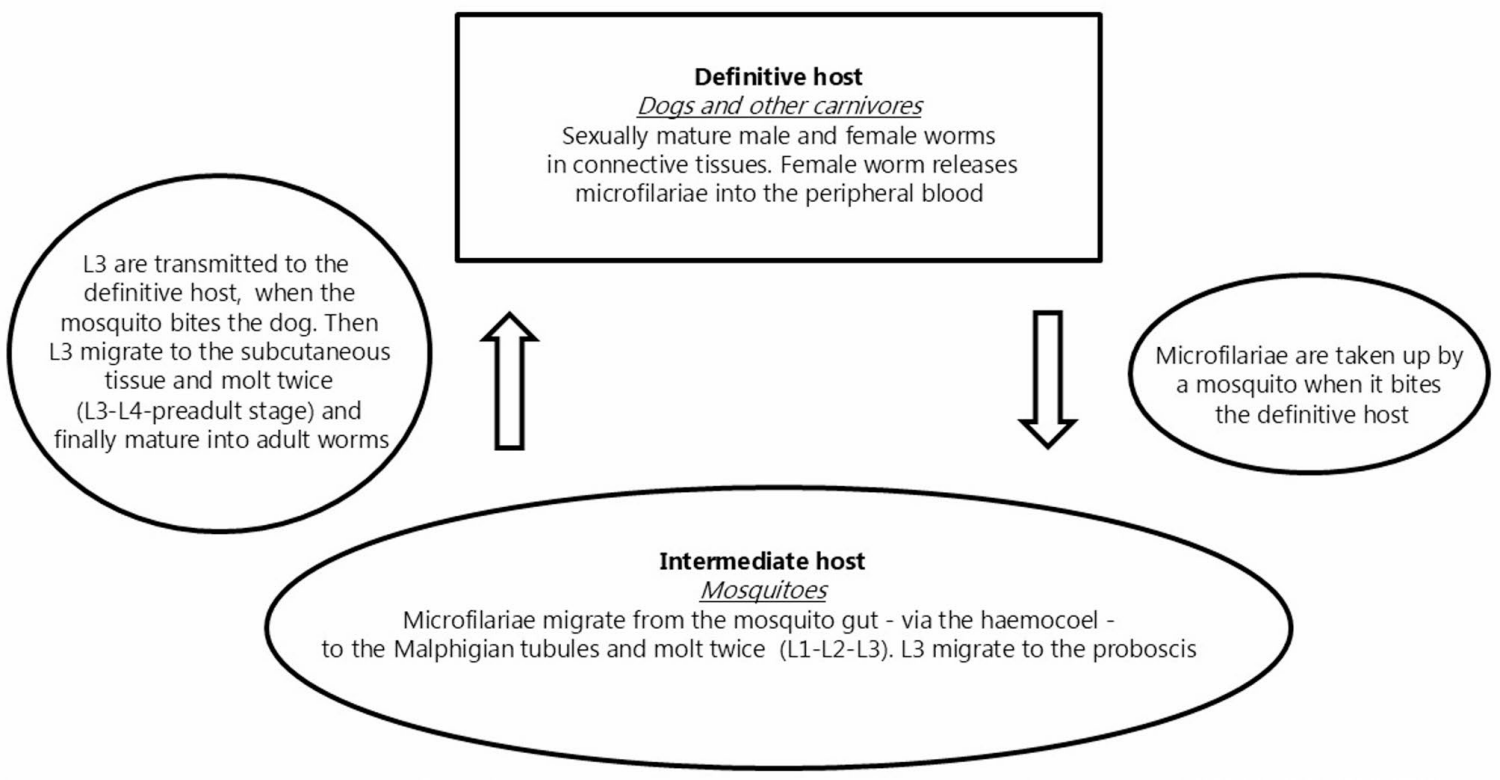
Diagrammatic view of the life cycle of *Dirofilaria repens*. The adult male and female worms reside in subcutaneous tissues of the dog. The viviparous females contain embryonated eggs from which fully developed microfilariae L1 are released to the blood stream. The microfilariae circulate in the vascular system. Vector mosquitoes bite the dog and take up L1. In the vector the larvae molt twice (L1-L2-L3) to reach the infective L3 stage, which migrate to the mosquito mouth parts and infect the dog, when the mosquito bites the host. The L3 larvae then migrate to the subcutaneous skin and molt (L3-L4-L5) and develop into the adult stage

*D. repens* is transmitted by vector mosquitoes and matures in dogs (Fig. 4). The finding in Denmark of a dog infected by a fully mature female *D. repens* carrying fully developed microfilariae *in uteri* indicates that import of dogs may represent a risk of introducing parasites of zoonotic and veterinary importance. The prepatent period in the dog is 189–239 days [36] and the parasite lifespan is several years [7]. Therefore, on a theoretical basis it cannot be excluded that the dog had been infected in Denmark following its import 15 months earlier (July 2023), a month with well described mosquito activity [37]. This would leave a possibility for local transmission, infection and development from third stage larva to the adult stage in the dog. When present in a dog in Denmark, the parasite species will have the possibility to colonize the region, where suitable vectors are present. This parasite species has already been reported from neighbouring countries (Finland, Estonia, Latvia, Lithuania, Poland, Germany), in which autochthonous transmission is known to occur [24]. Field studies and a series of experimental infections have documented that species within the dipteran genera *Aedes*,* Anopheles Coquillettidia* and *Culex* are suitable vectors [1, 2]. Thus, it is noteworthy that more than 30 mosquito species belonging to these four genera are endemic in Denmark [37, 38]. Behaviour of dogs in Central and Northern Europe (staying indoor overnight) may limit transmission in these regions compared to Southern Europe, where many dogs may stay outside [20]. Larval development in the vector is temperature dependent [1, 2, 8, 39] and therefore climatic conditions are considered to limit maturing and transmission in northern countries. However, transmission takes place in countries with corresponding climate conditions and borders to Denmark. The average air temperature in the Baltic countries is reported between 6.0 and 6.5 °C [24], which evidently appears to be sufficient for establishment of the *D. repens* life cycle. It is therefore noteworthy that the mean temperature at the present location for *D. repens* infection of a dog (Danish island of Bornholm) is even higher (8.5 °C), and further temperature elevations with foreseen climate changes are expected [40]. Based on the notion that the development (from microfilariae L1 to infective L3 larvae) in the vector requires at least 130 larval development units (LDU), it was calculated that successful establishment could take place in various locations in Germany even with marked daily temperature fluctuations [41]. The unit allows calculations of the time period needed at different temperature levels. This approach considers that northern regions exhibit marked seasonal and diurnal temperature variations, which influence larval development in the mosquito vector (poikilothermic insects and thereby dependent on the environmental temperature) [41]. The presence of suitable vectors and climatic conditions in Denmark, therefore, points to a risk for *D. repens* life cycle establishment. This was already framed [27] with regard to Danish conditions in general, and the present observation suggests that the risk may not be lower in the Baltic island of Bornholm. It is therefore relevant to increase focus on possible public health concerns, prevention strategies for pet owners, and recommendations for veterinary practitioners, including methods for isolation and diagnosis.

## Data Availability

The datasets used and/or analyzed during the current study are available from the corresponding author on reasonable request. GenBank (NCBI) accession nos. of the *Dirofilaria repens* sequences obtained in this study are: (PQ781236) (ITS) and (PQ772105) (COX1).

## Declarations

### Prior publication

Data have not been published previously.

### Authors’ information

KB is a parasitologist and immunologist with 40 years’ experience. PWK is a cell biologist with 30 years’ experience in molecular work. PJ has more than 30 years’ experience in veterinary practice.

## Abbreviations

COX1: Cytochrome c oxidase subunit 1
ITS: Internal transcribed spacer
mtDNA: Mitochondrial DNA
NADH: Nicotinamide adenine dinucleotide hydrogen
PCR: Polymerase chain reaction
rDNA: Ribosomal DNA
